# Clinicopathological characteristics of resected adenosquamous cell carcinoma of the lung: Risk of coexistent double cancer

**DOI:** 10.1186/1749-8090-5-92

**Published:** 2010-10-29

**Authors:** Hidetaka Uramoto, Sohsuke Yamada, Takeshi Hanagiri

**Affiliations:** 1 Second Department of Surgery, School of Medicine, University of Occupational and Environmental Health, Kitakyushu, Japan; 2 Department of Pathology and Cell Biology, School of Medicine, University of Occupational and Environmental Health, Kitakyushu, Japan

## Abstract

**Background:**

adenosquamous carcinoma (ADSQ) of non-small cell lung cancer (NSCLC) is a rare disease and the biological behavior and clinicopathological characteristics have not yet been thoroughly described.

**Method:**

This study reviewed the patient charts of 11 (1.6%) ADSQ cases among 779 patients with primary lung cancer who underwent a lung resection. The characteristics and clinicopathological factors were evaluated retrospectively.

**Results:**

Six of the 11 patients with ADSQ were male and five were female. The mean age was 67.3 years' olds. Three patients had pathological stage IA, one patient each had stage IB and IIA, five patients had stage IIIA, and one patient stage IIIB. Five patients had coexistent double cancer including 2 gastric, 1 rectal, 1 prostate and 1 bladder cancer. ADSQ was found less frequently in males than squamous cell carcinoma (SQ). ADSQ was found more frequently in older patients, with advanced stage, advanced T status, and lymph node metastases than adenocarcinoma (AD). The proportion with coexistent double cancer of AD, SQ, and ADSQ were 21.1, 17.6, and 45.5%, respectively. ADSQ had a significantly correlation with double cancer (ADSQ vs. non- ADSQ p = 0.03). A multivariate analysis showed no significant prognostic difference between the patients with ADSQ and non- ADSQ.

**Conclusions:**

In this study, cases with ADSQ showed no significantly prognostic difference in comparison to AD and SQ. However, surgeons must be cautious of any coexistent double cancer because approximately half of all patients with ADSQ of the lung have double cancer.

## Introduction

Lung cancer remains the most common cause of cancer death worldwide [1]. Despite recent improvements in diagnostic technologies, more than 50% of patients present locally advanced or distant metastatic disease, and the prognosis still remains unsatisfactory [2,3]. One reason for this is that the specific characterization of phenotype according to the pathological diagnosis has not been clarified, especially with regard to minor phenotypes, such as ADSQ [4] and large cell neuroendocrine carcinoma [5]. There are few reports of the clinical significance of ADSQ [6-9]. The effectiveness of surgical resection of ADSQ of the lung remains poorly defined because of the relatively low frequency of this type of tumor [6,7]. Shimizu. et al. reported that the survival rates for patients with ADSQ were statistically worse than for patients with SQ and AD, due to the highly aggressive pathological stage of ADSQ [7]. On the other hand, Hsia et al. reported that the prognostic behavior of ADSQ had no influence on survival in comparison to the survival curves with those for SQ and AD [4] consistent with previous reports [8,9]. Therefore, the prognostic impact of ADSQ in lung cancer still remains controversial. Furthermore, no series has addressed the correlation between the ADSQ and coexistent double cancer. This study retrospectively reviewed cases a 13-year period to clarify the correlation between the ADSQ and clinicopathological factors including coexistent double cancer and to elucidate the prognostic significance of ADSQ of the lung in comparison to the AD and SQ.

## Materials and methods

### Patients and methods

The characteristics and clinicopathological factors were evaluated retrospectively. Seven hundred and seventy-nine patients with primary lung cancer underwent a surgical resection between April 1994 to March 2006 at the Second Department of Surgery at University of Occupational and Environmental Health, Kitakyushu, Japan. Of these, 403, 187, 39, 24, 11, 125, and 10 patients were diagnosed as AD, SQ, large cell carcinoma, small cell carcinoma, ADSQ, multiple primary lung cancer (MPLC), and others, respectively. According to the established criteria, on routine microscopic examination using hematoxylin and eosin staining, a sample from a tumor containing at least 20% each of AD and SQ is classified as ADSQ [10]. Specimens from each of these patients were reviewed critically by one (S.Y.) pathologist. This study evaluated 11 cases (1.6%) with ADSQ among 779 resected primary lung cancers. The characteristics of the patients with ADSQ are shown in Table 1. They included 6 males and 5 females with a mean age of 67.3 years, ranging in age from 48 to 79 years. Of these eleven patients, all had undergone complete resections, and six were N0 cases. The pathological stages of the cancer were stage IA in 3 patients, IB in 1, IIA in 1, IIIA in 5, IIIB in 1, according to the TNM classification revised by the International union Against Cancer [11]. Patients were followed up with clinical assessments, chest X-rays, and tumor marker every month during the first year and every 3 months thereafter after operation. Computed tomography, bone scintigram and brain magnetic resonance imaging were all performed every 6 months. The mean observation period was 46.1 months.

**Table 1:** Characteristics of the patients with ADSQ

Case	Age	Sex	Symptom	Smoking	P-Stage	operation	Additional treatment
1	61	M	none	current	IIIA	L	carboplatin/paclitaxel
2	68	F	cough, fever	current	IIIA	L	carboplatin/paclitaxel
3	62	M	cough	never	IIIB	P	n.d.
4	72	F	cough	Former ^a^	IB	L	n.d.
5	77	F	none	current	IA	L	n.d.
6	79	M	none	ever	IA	L	n.d.
7	55	M	cough	current	IA	L	n.d.
8	72	M	none	current	IIIA	L	uracil and tegafur
9	74	F	none	never	IIIA	L	radiation
10	72	M	cough	current	IIA	L	n.d.
11	48	F	hemosputum	never	IIIA	L	carboplatin/paclitaxel

**Case**	**Recurrence**	**Chemotherapy for recurrence**	**Disease free interval (days)**	**Double ca**	**Outcome**	**Cause of death**	

1	+	n.d.	204	-	Dead	Lung cancer	
2	-	-	-	-	Alive	-	
3	-	-	-	Prostate ca **	Alive	-	
4	-	-	-	Gastric ca **	Alive	-	
5	-	-	-	Gastric ca *	Alive	-	
6	+	n.d.	734	Bladder ca*	Alive	-	
7	-	-	-	-	Alive	-	
8	+	cisplatin/5-Fu	145	-	Dead	Lung cancer	
9	+	carboplatin/uracil and tegafur	101	-	Dead	Lung cancer	
10	+	n.d.	995	Rectal ca*	Dead	Lung cancer	
11	+	carboplatin/docetaxel→cisplatin/5-Fu	192	-	Dead	Lung cancer	

^a ^Former smokers are defined persons who stopped smoking more than 3 years previously. L: Lobectomy, P: Pneumonectomy. n.d: not done.

* Double cancer was detected before treatment of lung cancer. ** It was done after that

### Statistical analysis

Statistical analyses were performed using the Stat view 5.0 software program for Windows (Abacus Concepts, Inc., California). The chi-square test was used to assess the relationship between ADSQ and each of the clinicopathological features. In the prognostic analysis, the Kaplan-Meier method was used to estimate the probability of survival, and survival differences were analyzed by the log-rank test. A multivariate analysis was performed according to Cox's proportional hazards model. A p-value less than 0.05 was considered to be significant.

## Results

### The clinical features of ADSQ patients

The clinical features of the patients with ADSQ are summarized in Table 1. The most frequent symptoms were cough. They included 8 smokers and 3 never smokers. Bronchoscopic examination indicated that the tumor was centrally located in two cases and was peripherally located in nine cases. All patients underwent bronchoscopy, and the results of biopsy or cytology revealed a malignancy in 7 (63.6%). The preoperative pathological diagnosis was SQ in three patients, non-small cell lung carcinoma in two patients and AD in one patient. The preoperative cytological diagnosis without definite pathological diagnosis was AD in one patient. All patients underwent a lobectomy except for one pneumonectomy. Five of 11 patients received postoperative adjuvant therapy including chemotherapy in 4 patients and radiation in one patient. Six patients had recurrent disease after surgery and the mean disease free interval was 56.5 months. Three of the 6 patients received chemotherapy treatment. Five of 11 (45.5%) patients had coexistent double cancer. Two patients had gastric cancer, and one patient each had prostate, bladder, and rectal cancer, respectively. Five patients died due to a recurrence of lung cancer. Complete follow-up data were available on all patients.

### Histological type between the primary lesion and lymph node metastasis

The prevalent histological differentiation of AD was moderately differentiated (45%, 5/11), while the prevalent SQ was moderately differentiated in 82% (9/11; Table 2). The two elements identified were either in contiguous fields or intermingling, and the ratios between the two yielded three groups. In 3 tumors, the AD component exceeded the SQ component. In 7 tumors, the reverse was true. Only one case showed both components present in roughly equal quantities. Lymph node metastases were found in 6 cases (55%) and were more frequent in cases with a larger SQ component in the primary lesion (Table 3).

**Table 2:** Degree of histological differentiation for each component

AD component	SQ component	No of cases	Total
Well	Well	1	
	Moderate	3	4
	Poor	0	

Moderate	Well	0	
	Moderate	5	5
	Poor	0	

Poor	Well	0	
	Moderate	1	2
	Poor	1	

**Table 3:** Comparison of Histological type between the primary lesion and lymph node metastasis

Primary lesion	No of cases	N0	lymph node metastasis		
			
			AD	ADSQ	SQ
AD > SQ	3	2	0	1*	0
AD = SQ	1	0	1	0	0
SQ > AD	7	3	1	1	2
Total	11	5	2	2	2

* Histological type of hilar and mediastinal lymph node metastasis was ADand ADSQ, respectively.

### Relationship between ADSQ and clinicopathological factors

When the clinicopathological factors were compared by AD, the status of ADSQ did not significantly affect the sex and coexistent double cancer. However, an ADSQ was found more frequently in old age, advanced stage, advanced T status, and N status. When the clinicopathological factors were compared by SQ, the status of ADSQ did not significantly affect the age, pathological stage, T status or N status. However, an ADSQ was found more frequently in females and coexistent double cancers (Table 4). ADSQ also showed a significant correlation with coexistent double cancers (ADSQ vs. non- ADSQ p = 0.03).

**Table 4:** Relationships between the level of ADSQ and the clinicopathological characteristics in 601 lung cancer patients.

Characteristics	Total	ADSQ	AD	SQ	**P value**^**a**^
Total	601	11	403	187	
Gender					
Male	398	6 (54.5)	226 (56.1)	166 (88.8)	
Female	203	5 (45.5)	177 (43.9)	21 (11.2)	0.9194/0.0011
Age (y)					
<70	286	5 (45.5)	210 (52.1)	71 (38.0)	
≥70	315	6 (54.5)	193 (47.9)	116 (62.0)	0.017/0.6198
Pathologic stage					
I-II	460	5 (45.5)	338(83.8)	117 (62.6)	
III-IV	141	6 (54.5)	65 (16.1)	70 (37.4)	< 0.01/0.2567
pT					
T1-2	521	6 (54.5)	375 (93.1)	140 (74.9)	
T3-4	80	5 (45.5)	28 (6.9)	47 (25.1)	< 0.0001/0.1367
PN					
N0	411	5 (45.5)	313 (77.7)	93 (49.7)	
N1-2	190	6 (54.5)	90 (22.3)	94 (52.2)	0.013/0.7564
Double ca					
Yes	123	5 (45.5)	85 (21.1)	33 (17.6)	0.053/0.023
No	478	6 (54.5)	318 (78.9)	154 (82.3)	

^a ^The p value of former before slashdot was calculated by between ADSQ and AD. The p value of latter after slashdot was calculated by between ADSQ and SQ.

### Influence of ADSQ on overall survival

A univariate analysis showed no significant prognostic difference between the patients with old age, histological type divided into ADSQ, coexistent double cancer, and MPLC. Meanwhile males, advanced T, and N status were associated with an unfavorable prognosis (Table 5). The five-year survival of AD, SQ, and ADSQ was 71.4, 55.8, and 50.6%, respectively. A multivariate analysis included such factors as sex, age, T status, N status, histological type, coexistent double cancer, and MPLC. Though, no significant prognostic difference was identified between the patients with regard to histological type, coexistent double cancer, and MPLC, the prognosis for patients with male sex, old age, advanced T, and N status was significantly worse, compared with the patients for counter features (Table 6).

**Table 5:** Univariate analysis using a proportional hazard model for the survival of the 799 lung cancer patients.

	Univariate analysis		
	
Variable	95% CI	Hazard ratio	P value
Gender			
Female		1	
Male	1.43-2.63	1.950	< 0.0001
Age (y)			
<70		1	
≥70	1.80-1.793	1.392	0.105
pT			
T1-2		1	
T3-4	1.86-3.27	2.46	< 0.0001
pN			
N0		1	
N1-2	2.25-3.73	2.90	< 0.0001
Histological type			
Non- ADSQ		1	
ADSQ	0.48-350	1.302	0.601
Double ca			
No		1	
Yes	0.61-1.14	0.833	0.253
MPLC			
No		1	
Yes	0.54-1.49	0.89	0.671

95% CI: 95% confidence interval.

**Table 6:** Multivariate analyses of various prognostic factors.

	Characteristics				
				
Variable	Unfavorable	Favorable	Risk ratio	95% CI	P value
Gender	Male	Female	1.81	0.24-2.47	< 0.001
Age (y)	≥70	<70	1.56	1.19-2.03	< 0.01
T status	3-4	1-2	1.97	1.47-02.64	< 0.0001
N status	Positive	Negative	2.62	2.02-3.39	< 0.0001
Histological type	ADSQ	Non- ADSQ	0.98	0.36-2.68	0.97
Double cancer	Yes	No	1.24	0.90-1.71	0.19
MPLC	Yes	No	1.15	0.69-1.93	0.58

## Discussion

The classification of lung carcinoma into a small cell lung carcinoma (SCLC) and NSCLC is highly reproducible. However, there are few studies about ADSQ with anecdotal reports on the subtype of ADSQ. ADSQ of the lung is a subset of pulmonary carcinomas, and comprises less than 4% of lung carcinomas [4,6-8]. It is quite difficult to diagnose ADSQ preoperatively [12]. In fact, only seven of 11 patients were diagnosed NSCLC as either AD and SQ. Interestingly, the preoperative pathological diagnosis was judged to the AD in case 1, nevertheless the preoperative cytological diagnosis was SQ by washing and brushing cytology under bronchoscopy. Physicians should therefore suspect ADSQ in cases where there is this type of preoperative mixed diagnosis.

This study identified no significant prognostic difference was identified between the patients with ADSQ and non-ADSQ. These findings are consistent with Hsia's data [4]. However, other researchers report the highly aggressive behavior of ADSQ [6,7,12-14]. This discrepancy might be attributed to three main factors. The first is that the current study observed very few patients with ADSQ. Due to the infrequent occurrence of this disease, no series reported to date has been of adequate size for a definitive statistical analysis. The second possibility is a previous international discrepancy in the pathological recording. At the present, Japanese lung cancer society defines ADSQ as tumor composed of SQ and AD components of at least 20% each [10]. However, previous World Health Organization histological classifications did not refer to the ratio of these two components and recent classification has required a minimum of 10% of each component. The third possibility may be the discrepancy among the pathological diagnosis [15].

This is the first report to address the problem of coexistent double cancer with ADSQ. In general, the prevalence of coexistent double cancer for the patients with lung cancer is about 10% of lung carcinomas [16]. Surprisingly, almost half of all patients with ADSQ of the lung were observed to have coexistent double cancer. Some clinical experiences have been reported [17] based on the concept of filed carcinogenesis in specific organ system such as respiratory or gastrointestinal tract, which are consistent with the current. Thus, behavior of tumor with ADSQ might be amore aggressive than the tumor with AD or SQ. It is necessary to suspect the presence of double cancer not only in the medical examination for the patients before treatment of ADSQ, but also during the follow-up after a complete resection.

Carcinogenesis is a multi-step gene abnormality and requires a certain amount of time to progress. ADSQ was found more frequently in old age than AD in this study suggesting that age is the one of the main risk factors for this type of cancer. Heterogeneity is frequent in cancer and ADSQ is an atypical heterogeneous tumor containing two distinct components of AD and SQ. Therefore, the current findings, which show a high prevalence of coexistent double cancer, suggest the cancer cells not only consist of mixture of AD and SQ, but also show progression of carcinogenesis with specific mechanisms in separate organs. The original histogenesis remains unclear. However, the biological impact is very interesting because the squamous cell component does not exist in the peripheral lung. Lymph node metastases were more frequent in cases with a larger SQ component in the primary lesion in this study. Therefore characterization of ADSQ may be a first step based on carcinogenesis to resolve the mystery of the heterogeneity. The histogenesis of ADSQ includes some possibilities including AD with squamous metaplasia, collision tumor, high-grade mucoepidermoid carcinoma, and bi-potential undifferentiated cell origin [18]. Niho et al. showed that SQ and AD components showed identical monoclonal patterns, suggesting that the SQ and AD components originate from the same cell [19]. Kanazawa et al. also reported the clonal expansion in the carcinogenic process by the monoclonal transition from SQ to AD in ADSQ [20]. Therefore, the histogenesis of ADSQ might represent a phase in ongoing differentiation from a common cancer stem cell.

In conclusion, this current study demonstrated that ADSQ is an unusual histological subtype of NSCLC and approximately half of the patients with ADSQ had coexistent double cancer, thus suggesting that cancer cells in ADSQ demonstrate progressive carcinogenesis with specific mechanisms.

## Abbreviations

ADSQ: adenosquamous carcinoma; SQ: squamous cell carcinoma; AD: adenocarcinoma; NSCLC: non small cell lung cancer; SCLC: small cell lung carcinoma; MPLC: multiple primary lung cancer; 95% CI: 95% confidence interval.

## Competing interests

The authors declare that they have no competing interests.

## Authors' contributions

This report reflects the opinion of the authors and does not represent the official position of any institution or sponsor. The contributions of each of the authors were as follows:

HU were responsible for reviewing previous research, journal handsearching, drafting report. SY was responsible for pathological data. TH was responsible for quality checking and data processing. HU was responsible for project coordination. All authors have read and approved the final manuscript.
